# Risk perception and travel satisfaction associated with the use of public transport in the time of COVID-19. The case of Turin, Italy

**DOI:** 10.1371/journal.pone.0265245

**Published:** 2022-03-31

**Authors:** Martina Gnerre, Daniela Abati, Manuela Bina, Federica Confalonieri, Silvia De Battisti, Federica Biassoni

**Affiliations:** Catholic University of the Sacred Heart, Milan, Italy; The University of Tokyo, JAPAN

## Abstract

The present study examined the association between risk perception and travel satisfaction related to the use of public transport (PT) during COVID-19 pandemic in Turin, Italy. A total of 448 PT users took part in an online survey conducted from January to March 2021. It investigated safety and risk perception related to the use of PT, and the users’ subjective experience, measured through the Satisfaction with Travel Scale (STS). These perceptions were compared for three time scenarios: before the pandemic, during the pandemic, and in the future at the end of the pandemic emergency. Results showed that COVID-19 influenced respondents risk perception both during the pandemic and in their projections about the future, especially for females. The risk of contagion from COVID-19 is perceived as higher inside a PT vehicle than in the adjacent/waiting spaces. Regarding travel satisfaction, the overall scores of the STS indicated that the pandemic has impacted reported well-being while travelling, both now and in the future. The dimension of activation shifted towards the negative pole and did not indicate a return to risk perception before the pandemic levels at the end of the crisis (especially for females). Respondents reported a significant decrease in their level of pleasure and satisfaction during the pandemic, but expect that in the future these levels will go back to the levels previously experienced. Regarding travel satisfaction, PT users aged 36 to 50 years reported the highest level of satisfaction, while younger users (18 to 35 years) reported the lowest degree of satisfaction in all three time scenarios. Overall, the results clearly pinpoint that health-related perceived risk is becoming a key determinant for PT use. Within this context, different dimensions of travel satisfaction proved to be impacted differently by the pandemic, for both current and future scenarios.

## 1. Introduction

On 11 March 2020, the World Health Organization (WHO) [1] announced SARS-CoV-2 (COVID-19) as a global emergency. More than a year on, the pandemic continues to have critical social, economic and psychological implications. A key measure used to control the spread of the contagion has been social distancing. As such, many governments, including the Italian government, have prohibited people from travelling unless absolutely necessary. As a consequence of such measures the demand for public transport (PT) has dramatically decreased. In addition, PT accessibility has undergone a major reorganisation based on specific indications, recommendations and restrictions [2–4]. The significant reduction in demand for PT also originated from self-imposed restrictions related to people’s fear of contagion [4]. PT users’ subjective perceptions of safety criteria have changed, resulting in a shift in mobility choices towards an increased reliance on private cars [5,6], a trend that is strongly inconsistent with the sustainability policies adopted by cities in the European Union [7,8]. In a survey conducted in ten countries about mobility during the COVID-19 emergency, the authors found that the major part of the Italian respondents modified their mobility habits reducing any of the transport modes [9]. Hence, there is considerable interest in the relationship between mobility behaviours and COVID-19. The changes in travel patterns due to the advent of the COVID-19 pandemic have been investigated by focusing on different variables, such as age, gender or lifestyle choices [4,6,8]. This study adds to previous research by analysing if and how the pandemic has affected risk perception and satisfaction with travel on PT, considering the role of gender and age. The presented research was conducted in a large city in northern Italy, Turin, which has PT system that includes 80 bus lines, 8 tram lines and 1 metro line. The study coincided with a period of mass restrictions imposed on PT use to increase public safety in response to COVID-19. These restrictions included reduced occupancy of PT vehicles, stringent social distancing measures and the requirement to wear face masks.

### 1.1 Public transport use and risk perception

Rosa [10] outlines risk as ‘a situation or an event where something of human value (including humans themselves) is at stake and where the outcome is uncertain’ (p. 56). Everyone interprets potential risk differently, so risk perception is a subjective evaluation of the probability of a specific type of accident happening and how involved we are with the consequences [11]. Risk perception is a multidimensional construct influenced by several elements, including the sources of risk, novelty and knowledge about the hazard. In addition, the way respondents estimate probability judgements and affective reactions are shaped by factors such as attitudes, behaviours and culture [11–13]. However, worry is considered the most important predictor of risk perception [14]. In relation to transport, worry has been studied primarily in relation to the possibility of a crash occurring. Moreover, research highlights that when evaluating risk in relation to transport, people usually underestimate the risks because of the perceived utility of transport, and the level of knowledge and control they assume to have [15]. Moen and colleagues [14] showed a difference between the evaluation of risk related to transport when people perceive themselves as being in control (e.g. private cars) in comparison to a perception of less control (e.g. plane, train, bus, boat or ferry). This means that the perceived control for PT users is lower than for users of private cars. Consequently, it is expected that perceived control may influence the use of PT versus private modes of mobility as well. Risk perceptions related to individual safety, in terms of health, accidents or crime, may affect individuals’ choice of travel mode or trip frequency; a current example is the risks associated with COVID-19, related perceptions of safety and preference for private modes of transportation [16]. Before the COVID-19 pandemic, many studies investigated user perception of risk and the relative impact on their use of PT. For instance, Delbosc and Currie [17] found that the most significant factors impacting risk perception of PT, are users’ trust in others and how safe they feel at home and on the street at night. A recent study highlighted risk perception associated to the use of PT in terms of personal safety (e.g. being alone in a concealed space with strangers) [18]. Other researches focused on risk perception in relation to fear of crime; work by Thevasthan and Balachandran [19] considered passenger perception of safety as one of the most important factors affecting their PT service experience. Furthermore, the role of age and gender in risk perception associated to PT use has been considered and both age and gender have been proved to influence risk perception in the use of different travel modes. Studies have argued that women are more worried about risk in relation to travel and are therefore more likely to avoid certain travel choices [18,20,21]. Shelat et al. [22] found that both older and female travellers showed more consciousness and higher risk perception in relation to PT. Conversely, Borkowski et al. [4], did not find any influence on risk perception of age or gender, only of occupation. An overview of literature related to private transportation showed that younger respondents reported lower levels of perceived risk than older respondents [23–25], while women reported more risk than men [25,26]. Moen et al. [14] explored risk perception of crashes and found that females expressed higher levels of worry with regard to both public and private mobility. However, research by Delbosc et al. [17], focused on risk perception of crime, argued that perceived risk was not directly explained by gender.

Research on risk perception in relation to COVID-19 has highlighted that women present higher levels of risk perception than men [27], and older people estimate lower levels of risk than younger people [28]. To date few research has focused on the perception of risk associated with PT in the context of COVID -19 [29–31], despite there are several documents by organizations like The International Association of Public Transport (UITP) [32,33] and The International Union of Railways (UIC) [34] addressing this issue.

Nonetheless, the perceived risk of potential for infection and the attitude towards this risk have proved critical elements in determining the reduction of mobility during this pandemic emergency [2,4].

### 1.2. Satisfaction with travel, risk and safety perception

Travel satisfaction is defined here as a specific satisfaction measure connected to subjective well-being based on how travellers evaluate their daily travel experiences [35]. From a theoretical point of view, Ettema et al. [36] demonstrated that travel satisfaction for car drivers is influenced by their experience of safety. However, to date only a few studies have focused on travel satisfaction in relation to risk perception for urban PT service users [17,37,38], and even less in the context of COVID-19 as an emerging and ongoing risk factor [39].

As seen before, in existing studies, risk in relation to PT use is measured in terms of perceived possibility of being a victim of a crime (e.g. harassment or violence) or an accident (i.e. crash or fatality rates per number of trips) [40–43], and not in terms of subjective experience or psychological factors that may influence attitudes towards PT. Ettema [36] considers the subjective perception of personal safety in a trip as a ‘soft factor’ however with the arrival of the pandemic emergency the possibility of being infected may impact travel satisfaction (and hence the mobility choices) to become a ‘hard factor’.

Before the pandemic, previous studies conducted in the European context showed that users were generally satisfied with PT services, though satisfaction with daily travel differed significantly between urban areas [43,44]. These studies did not account for the risk of virus spread, possibly because the PT users did not pay much attention to this factor before COVID-19 [39]. In the COVID-19 context, the concept of travel satisfaction in relation to PT deserves investigation as a potential way to understand possible changes in the motivations, perceptions and evaluations of users. Prior to the pandemic, satisfaction was considered one of the key factors to improve the use and expansion of PT services [44], however, contagion-related risk perception has now become an additional and significant factor influencing mobility choices.

A number of studies argue that several socio-demographic variables are associated with different degrees of travel satisfaction [45–49]. One such variable is age, with older people generally showing higher travel satisfaction [50,51] and users in the middle-age categories more likely to be satisfied with their journey [49,50]. Gao [50] found that younger travellers report lower travel satisfaction levels for PT, while Zhang et al. [51] reports that younger passengers are often enthusiastic participants in the evaluation of bus services. Another variable is gender, with some research reporting that gender is not significantly related to satisfaction, while other studies indicate that gender is a significant influence [48,50]. Despite gender and age being identified as two important variables in transportation research, few studies have dealt with its impact on travel satisfaction in the context of COVID-19.

### 1.3. The present study

This study is part of a new line of research which aims to develop an understanding of the psychological mechanisms underlying mobility choices and behaviours during the COVID-19 pandemic and provide future projections in relation to this [16,39]. Specifically, this study focused on the impact of the COVID-19 pandemic on travel satisfaction and risk perception related to the use of PT. The objectives of the study were: (1) to investigate risk perception related to PT in terms of virus contagion; (2) to understand the impact of the COVID-19 pandemic on the satisfaction with travel of PT users; (3) to investigate the correlation between risk perception and the three dimensions of satisfaction with travel as measured by the Satisfaction with Travel Scale (STS) before, during and after pandemic; and (4) to explore the impact of the variables of gender and age on risk perception and satisfaction with travel of PT users in each of the three time scenarios.

These topics were investigated by collecting and comparing measures of risk perception and travel satisfaction with reference to three different time scenarios (past, present and future) and considering age and gender differences. Hence, the data collected was: retrospective; related to the present (during a period of mass mobility restrictions and exceptional measures for PT); and related to future expectations.

In a period of pandemic and for post-pandemic projections, providing mobility that is safe from a health perspective while continuing to support sustainable modes of transport, is a central challenge for PT agencies and all stakeholders involved in health and transport. Within this framework, understanding users’ satisfaction with travel and users’ risk perception, are key factors for transport planning, service improvement and effective communication to address PT users’ needs in relation to information and reassurance.

## 2. Method

### 2.1. Procedure

The study was conducted using a self-administered, cross-sectional online survey among a total of 517 unpaid volunteers who were all users of PT in Turin, a metropolitan area of northern Italy. Participants were recruited through local media advertisements with an invitation to participate in a COVID-19 and mobility choice online survey. Of the participants in the initial sample, 69 (13%) did not complete the questionnaire or were excluded according to predefined exclusion criteria. Subjects who were not regular or occasional users of PT in Turin metropolitan area were excluded. PT users under 18 were also excluded because they needed a special consent from their parents to take part in the survey and we did not have the possibility to check for such permission appropriately. Moreover, we chose to not include participants over 70 years old, since they were strongly recommended not to use PT unless necessary during the considered period. Hence, the sample ranges from 18 to 70.

The final sample included 448 participants divided into three age groups: 39% aged 18 to 35 years; 32% aged 36 to 50 years; and 29% aged 51 to 70 years. Across these age groups, 57% were women, 42% men and 1% chose to not specify. All demographic characteristics for this study are presented in Table 1.

**Table 1:** Demographic characteristics of the participants.

Participants’ characteristics	Percentage
**Age**	
18–35	39%
36–50	32%
51–70	29%
**Gender**	
F	57%
M	42%
Gender diverse	1%
**Residence**	
Turin	92.45%
Other nearby provinces	8.12%
**Work**	
Employee	67%
Unemployed	8%
Retired/homemaker	2%
Freelance	6%
Student	14%
Other	3%
**Working mode**	
Working from home	18%
50% working from home and 50% working from office	32%
Recently working from office	21%
Always working from office	25%
Layoff	1%
Other	3%

The responses were collected over a period of seven weeks from January to March, 2021, during a period where mass restrictions for PT passengers were imposed to increase safety (e.g. reduced occupancy of vehicles, stringent social distancing measures and the requirement to wear face masks).

Survey response time was monitored to flag any respondents who did not spend an adequate amount of time answering the questions. Prior to completing the survey respondents were asked to read a written introduction explaining the study, and to provide their informed consent. The collected data was analysed anonymously. The study was approved by the local ethics committee and conducted according to the Declaration of Helsinki, and informed consent was obtained from every participant.

### 2.2. Instruments

The survey took approximately 15 minutes to complete, comprised of four sections with most responses expressed using a Likert-scale. The first section of the survey aimed to collect data about: socio-demographics; compliance with security rules aimed to protect PT users from COVID-19 transmission (e.g. social distancing and reduced physical contact); attitudes toward the use of face masks; and trust in the authorities.

The second section of the survey aimed to capture transport mode choice and moving habits. Seven options for mode of transport were provided: (1) Car, (2) PT, (3) Bike, (4) Bike sharing, (5) Car sharing and (6) Walking.

The third section of the survey investigated perceptions of risk and safety in the use of PT during the COVID-19 pandemic emergency in relation to the possibility of infection. Finally, the fourth section collected data about travel experiences as measured through the STS. Perceptions of risk, safety and travel experience were investigated for each of the time scenarios: before, during and after the pandemic. This paper focuses on the results of the third and fourth sections of the survey.

#### 2.2.1. Risk perception measures

To investigate risk perception in the use of PT, the survey asked participants: ‘How do you perceive the risk of being infected with COVID-19 while traveling on public transport?’ A version of this question was asked for each of the three temporal scenarios (before, during and after the pandemic). Respondents answered on a 7-point Likert scale ranging from 1 (no risk at all) to 7 (extremely risky).

To the best of our knowledge, there are no scales designed to measure health risk perception in relation to the use of PT. As such, a new risk perception measure was developed for this study and used to capture user perceptions during the COVID-19 outbreak. The scale employed was developed from an initial pool of 12 items identified within a literature review on the topic of risk perception investigated from a psychological point of view [11–13,15,21,24–28,52–54]. The pool of 12 items was revised and discussed by a group of six experts and resulted into the final version of the scale, comprising 10 items. The internal consistency of the scale was assessed using Cronbach’s alpha coefficient and proved satisfactory (α = 0.77). The scale was composed of two subscales. The first subscale referred to the experience of travelling on a PT vehicle, and the second to staying or moving in the adjacent/waiting spaces (see Tables 2 and 3). Specifically, the first subscale (composed of 4 items) was used to measure risk perception related to the experience of travelling inside a subway vehicle. A picture of a subway vehicle, equipped with stickers that remind the need to keep a safe distance due to the risk of contagion, was shown.

**Table 2:** The first subscale of the risk perception scale was used to measure risk perception related to the experience of traveling in a PT vehicle.

*How much do you perceive the risks of COVID-19 while traveling with PT in the peak hours*?
While I wait for the bus or the team
When I pass someone on the street who is not wearing a mask
When I pass someone on the street who is wearing a mask
While I’m waiting for the subway
When I wait to cross the street at a pedestrian crossing
While I walk out of the metro/train station

Totally disagree 1 2 3 4 5 6 7 Totally agree.

**Table 3:** The second subscale of risk perception.

I would feel safe in this situation in relation to the possibility of the COVID-19 contagion
If there were people standing positioned on the red signals, I would still feel safe from the possibility of COVID-19 contagion
I think that in peak hours it is possible to maintain a situation like the one pictured
It seems a very livable situation and I would like to take the subway always in these conditions

Not risky at all 1 2 3 4 5 6 7 Extremely risky.

Traveling in a subway was chosen as prototypic example of in-vehicle experience because of it being underground, possibly crowded and needing ventilation efficiency. Responses were provided on a 7-point Likert scale ranging from 1 (totally disagree) to 7 (totally agree). The second subscale (composed of 6 items) investigated risk perception related to the experience of staying or moving in the adjacent/waiting spaces. Responses were provided on a 7-point Likert scale ranging from 1 (no risk at all) to 7 (extremely risky).

#### 2.2.2 Travel satisfaction measures

Travel satisfaction in the three different time periods (before the pandemic, during the pandemic and after the pandemic) was measured using the STS, an instrument that measures customer service experience relating to travel without focusing on any particular travel mode.

STS consists of two affective dimensions and one cognitive dimension (see Table 4) [35]. These affective dimensions are activation or arousal (e.g. stressed or relaxed) and valence or pleasure (e.g. bored or engaged) based on the core affect approach by Russell [55] and the Swedish Core Affect Scale (SCAS) [56]. The three dimensions, one cognitive and two affective, are positively correlated constructs included in a higher-order measure of overall satisfaction with travel. Like the SCAS, the STS adopts two sets of three adjective pairs that are a combination of valence and activation (i.e. positive activation/negative deactivation and positive deactivation/negative activation), the respective adjective pairs are enthusiastic/bored, engaged/fed up alert/tired, calm/stressed, confident/worried and relaxed/hurried. The cognitive dimension relates to the perceived quality of service and is measured through a set of sentences regarding the travel experience (i.e. travel was the best/worst I can think of, travel was a high/low standard and travel worked out/did not work out well). The items in the STS are answered on a 7-point scale, ranging from negative (−3) to positive (3). The internal consistency (Cronbach’s alpha) of this scale varies from 0.84 to 0.88 [57] (as far as the present study is regarded, Cronbach’s alpha was calculated and resulted 0.84) and empirical studies have indicated the efficiency of this measure [35,36,58]. The STS has been validated extensively for PT and for other modes of transport [59–61].

**Table 4:** Statements included in the satisfaction with travel scale (STS).

**Negative activation**		**Positive de- activation**
Hurried Worried Stressed	-3–2–1 0 1 2 3	Relaxed Confident Calm
**Negative de-activation**		**Positive activation**
Tired Bored Fed up	-3–2–1 0 1 2 3	Alert Enthusiastic Engaged
**Negative Cognitive evaluation**		**Positive Cognitive evaluation**
Travel was worst I can think of Travel was low standard Travel did not work out well	-3–2–1 0 1 2 3	Travel was best I can think of Travel was high standard Travel worked out well

## 3. Results

### 3.1 Risk perception

**Fig 1:**
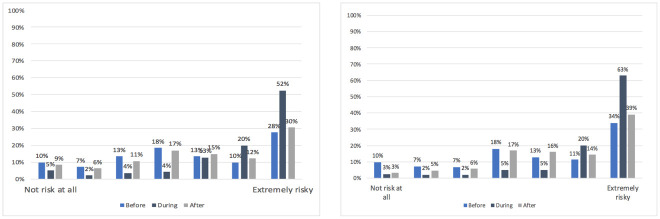
Distribution of risk perception evaluation over time, comparing responses of males (left) and females (right) to the question: ‘How much do you perceive the risk of being infected with COVID-19 while traveling with public transport?’.

**Fig 2:**
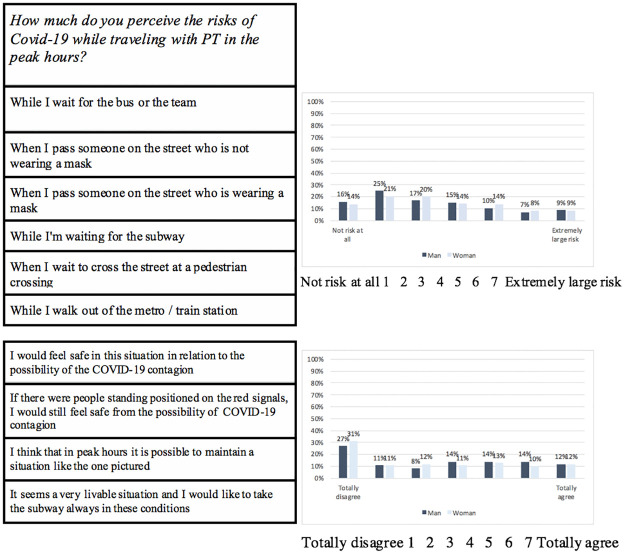
Distribution of risk perception evaluation for the two subscales. The distribution of each single item follows the distribution of each subscale.

The distribution of answers to the question: ‘How much do you perceive the risk of being infected with COVID-19 while traveling with the public transport?’, related to the three different time scenarios summarised in Fig 1. The mean values of risk perception evaluation were then calculated and a 2 (gender) × 3 (age group) × 3 (time scenario: before the pandemic vs during the pandemic vs after the pandemic) analysis of variance (ANOVA) with repeated measures on the last factor was performed to compare the mean values of risk perception. A main effect of the three time scenarios emerged [F (2,382) = 67.99, p < .001] and Tukey HSD (honestly significant difference) test confirmed the differences among all three temporal conditions (*p* < .05). Gender also proved to have a significant effect on risk perception [F (1,330) = 4.61, p < .05], with female PT users indicating higher levels of risk perception. A significant interaction effect of gender and temporal scenario was present only for the future scenario [F (3,334) = 3.12, p < .05], with female PT users declaring higher levels of risk perception. The demographic variable age showed no influence on participant perception of risk. The mean value of the items was calculated for each of the two risk perception subscales. Fig 2 shows the distribution of the mean values along the 7-point Likert scale. The perceived risk of COVID-19 infection was higher inside a PT vehicle than in the adjacent/waiting spaces [F (1,337) = 85.37, p < .05]. Univariate ANOVA was performed to analyse the effect of age or gender on the two risk perception evaluations (inside a vehicle vs in the adjacent/waiting spaces) and no significant effect emerged.

### 3.2. Travel satisfaction

A repeated measure ANOVA was performed to compare the global scores of travel satisfaction and each of the three dimensions of travel satisfaction in the three temporal scenarios. Regarding the global scores, a significant difference emerged [F (2,542) = 37.04, p ≤ .001]. Tukey HSD showed significant differences among all three temporal scenarios, with the highest levels of global travel satisfaction before the pandemic (p ≤ .05) and the lowest during the pandemic.

Considering each one of the STS dimensions separately, repeated measure ANOVA revealed that the dimension of activation was significantly different in the three time scenarios [F (2,546) = 61.57, p ≤ .001] and Tukey HSD showed significant differences among all three scenarios with the highest levels of activation during the pandemic and the lowest before the pandemic (p ≤ .01). With regard to the dimensions of pleasure and satisfaction, ANOVA showed a significant effect of the time-scenario (F (2,544) = 354, p ≤.05; F (2,544) = 1.83, p ≤ .001, respectively). For both pleasure and satisfaction, Tukey HSD revealed no significant differences between the conditions ‘before the pandemic’ and ‘after the pandemic’. But a significant difference was found between ‘before the pandemic’ and ‘during the pandemic’ and for ‘during the pandemic’ and ‘after the pandemic (p ≤ .05), with the highest levels of pleasure and satisfaction recorded for ‘before the pandemic’ (see Tables 5–7).

**Table 5:** Mean and standard deviation of the three dimensions and of the global score of STS before COVID-19.

	Males	Females	P-value	Young adults	Middle-aged adults	Older adults	P-value
**Activation**	0.37 (1.59)	0.09 (1.51)	.12	-0.90 (1.63)	0.50 (1.51)	0.21 (1.44)	.02
**Pleasure**	-0.10 (1.25)	-0.14 (1.20)	.75	-0.33 (1.20)	0.06 (1.14)	-0.18 (1.31)	.07
**Satisfaction**	0.06 (1.22)	- 0.22 (1.38)	.65	-0.44 (1.36)	0.28 (1.16)	-0.15 (1.32)	.00
**Global score**	0.10 (1.17)	- 0.9 (1.20)	2.07	-0.28 (1.21)	0.28 (1.10)	- 0.04 (1.19)	.003

**Table 6:** Mean and standard deviation of the three dimensions and of the global score of STS during COVID-19.

	Males	Females	P-value	Young adults	Middle-aged adults	Older adults	P-value
**Activation**	0.70 (1.44)	-0.99 (1.31)	.84	-0.81 (1.28)	-0.86 (1.51)	-0.93 (1.37)	.84
**Pleasure**	-0.29 (1.16)	-0.34 (0.96)	.70	-0.52 (0.99)	-0.20 (1)	-0.25(1.12)	.07
**Satisfaction**	-0.48 (1.22)	-0.64 (1.16)	.28	-0.74 (1.17)	-0.45 (1.12)	-0.51 (1.25)	.21
**Global score**	-0.49 (1.09)	-0.66 (0.94)	.16	-0.69 (0.96)	-0.52 (1)	-0.56 (1.05)	.48

**Table 7:** Mean and standard deviation of the three dimensions and of the global score of STS after COVID-19.

	Males	Females	P-value	Young adults	Middle-aged adults	Older adults	P-value
**Activation**	0.21 (1.51)	-0.31 (1.48)	.003	-0.24 (1.43)	-0.04 (1.56)	-0.04 (1.55)	.55
**Pleasure**	0.00 (1.37)	-0.19 (1.17)	.18	-0.31 (1.24)	-0.02 (1.21)	-0.03 (1.29)	.16
**Satisfaction**	-0.05 (1.33)	-0.33 (1.26)	.06	-0.49 (1.22)	-0.08(1.27)	-0.11 (1.37)	.04
**Global score**	0.50 (1.26)	-0.28 (1.18)	.01	-0.69 (0.96)	-0.52 (1)	-0.56 (1.05)	.48

In relation to the effect of gender, when considering the global score of the STS no effect emerged for the before and during pandemic scenarios. However, for the post-pandemic scenario the male subsample recorded higher rates of satisfaction than the female subsample [F (1,293) = 553, p ≤ .01]. When considering the three different dimensions of the STS separately, an interaction effect of gender and scenario emerged for activation, with males scoring higher rates of activation than females in the post-pandemic scenario [F (1,293) = 914, p ≤ .01]. Regarding the pre-pandemic and pandemic scenarios, no significant differences emerged between males and females for activation and no effect of gender emerged for pleasure and satisfaction.

The demographic variable of age did not have any significant effect on the global score of the STS, except for the period before the pandemic [F (2,301) = 597, p ≤ .1] with younger users recording lower scores for global satisfaction. Regarding activation, scenario showed a significant main effect [F (2,546) = 61,57, p ≤ .1], since activation level increased significantly during the pandemic. In addition, an interaction effect of age and time scenario emerged regarding activation [F (4,542) = 265, p < .5], with younger users significantly more activated than older users in the post-pandemic scenario. Regarding the satisfaction dimension, both scenario and age showed significant main effects [F (2,540) = 19.08, p ≤ .01 and F (2,540) = 4.59, p < .5, respectively]. Finally, age showed no significant effect on the pleasure dimension. A summary of the main and interaction effects of all variables reviewed in this study are shown in Tables 5–8.

**Table 8:** Effects of the variables time scenario, gender and age on risk perception (global and two subscales) and satisfaction with travel (global and three components).

Dimension	Time scenario	Gender	Age
Risk perception (global)	.001***	.16	.53
Risk perception (vehicle)	-	.14	.44
Risk perception (spaces)	-	.63	.23
Satisfaction with travel (global)	.001***	.41	.05*
Activation	.001***	.37	.03*
Pleasure	.03*	.68	.46
Satisfaction	.00***	.73	.03*

* is for p < .05,

** is for p < .01,

*** is for p < .001 (two-tailed test).

### 3.3. Correlations between risk perception and travel satisfaction

To further investigate the association between risk perception and the three dimensions of the STS in each of the three temporal scenarios, a correlation analysis using Pearson’s correlation coefficient (r) was performed. Risk perception and activation were found to be negatively correlated, despite their relationship being weak. The strength of the correlation increased from the risk perception before the pandemic (r (448) = -0.22, p < .001) to the risk perception during the pandemic (r (448) = -0.30, p < .001) and the risk perception after the pandemic (r (448) = -0.33, p < .001) (see Table 9). The correlations for pleasure and satisfaction were slightly weaker than with activation (Tables 10 and 11). The following values are related to the correlation between risk perception with pleasure and satisfaction dimensions, respectively, in the three temporal scenarios: before the pandemic (pleasure: r (448) = -0.12, p < .05; satisfaction: r (448) = -0.12, p < .05), during the pandemic (pleasure: r (448) = -0.30, p < .001; satisfaction: r (448) = -0.27, p < .001) and after the pandemic (pleasure: r (448) = -0.23, p < .001; satisfaction: r (448) = -.30, p < .001).

**Table 9:** Correlation matrix with Pearson’s correlations among risk-perception measures and STS activation dimension over time.

		**Risk perception before the pandemic**	**Risk perception during the pandemic**	**Risk perception after the pandemic**	**Activation before the pandemic**	**Activation during the pandemic**	**Activation after the pandemic**
**Risk perception before the pandemic**	Pearson’s r	—					
	p-value	—					
**Risk perception during the pandemic**	Pearson’s r	0.38	—				
	p-value	< .001	—				
**Risk perception after the pandemic**	Pearson’s r	0.69	0.67	—			
	p-value	< .001	< .001	—			
**Activation before the pandemic**	Pearson’s r	-0.22	-0.17	-0.30	—		
	p-value	< .001	0.002	< .001	—		
**Activation during the pandemic**	Pearson’s r	-0.14	-0.30	-0.22	0.33	—	
	p-value	0.017	< .001	< .001	< .001	—	
**Activation after the pandemic**	Pearson’s r	-0.25	-0.21	-0.33	0.49	0.43	—
	p-value	< .001	< .001	< .001	< .001	< .001	—

**Table 10:** Pearson’s correlation matrix among risk-perception measures and STS pleasure dimension over time.

		**Risk perception before the pandemic**	**Risk perception during the pandemic**	**Risk perception after the pandemic**	**Pleasure before the pandemic**	**Pleasure during the pandemic**	**Pleasure after the pandemic**
**Risk perception before the pandemic**	Pearson’s r	—					
	p-value	—					
**Risk perception during the pandemic**	Pearson’s r	0.38	—				
	p-value	< .001	—				
**Risk perception after the pandemic**	Pearson’s r	0.69	0.67	—			
	p-value	< .001	< .001	—			
**Pleasure before the pandemic**	Pearson’s r	-0.12	-0.18	-0.18	—		
	p-value	0.038	0.002	0.002	—		
**Pleasure during the pandemic**	Pearson’s r	-0.22	-0.30	-0.29	0.48	—	
	p-value	< .001	< .001	< .001	< .001	—	
**Pleasure after the pandemic**	Pearson’s r	-0.17	-0.21	-0.23	0.58	0.61	—
	p-value	0.004	< .001	< .001	< .001	< .001	—

**Table 11:** Correlation matrix with Pearson’s correlations among risk-perception measures and STS satisfaction dimension over time.

		**Risk perception before the pandemic**	**Risk perception during the pandemic**	**Risk perception after the pandemic**	**Satisfaction before the pandemic**	**Satisfaction during the pandemic**	**Satisfaction after the pandemic**
**Risk perception before the pandemic**	Pearson’s r	—					
	p-value	—					
**Risk perception during the pandemic**	Pearson’s r	0.38	—				
	p-value	< .001	—				
**Risk perception after the pandemic**	Pearson’s r	0.69	0.67	—			
	p-value	< .001	< .001	—			
**Satisfaction before the pandemic**	Pearson’s r	-0.12	-0.14	-0.23	—		
	p-value	0.031	0.013	< .001	—		
**Satisfaction during the pandemic**	Pearson’s r	-0.13	-0.27	-0.30	0.50	—	
	p-value	0.039	< .001	< .001	< .001	—	
**Satisfaction after the pandemic**	Pearson’s r	-0.21	-0.21	-0.30	0.58	0.57	—
	p-value	< .001	< .001	< .001	< .001	< .001	—

### 3.4 A mediation model

**Fig 3:**
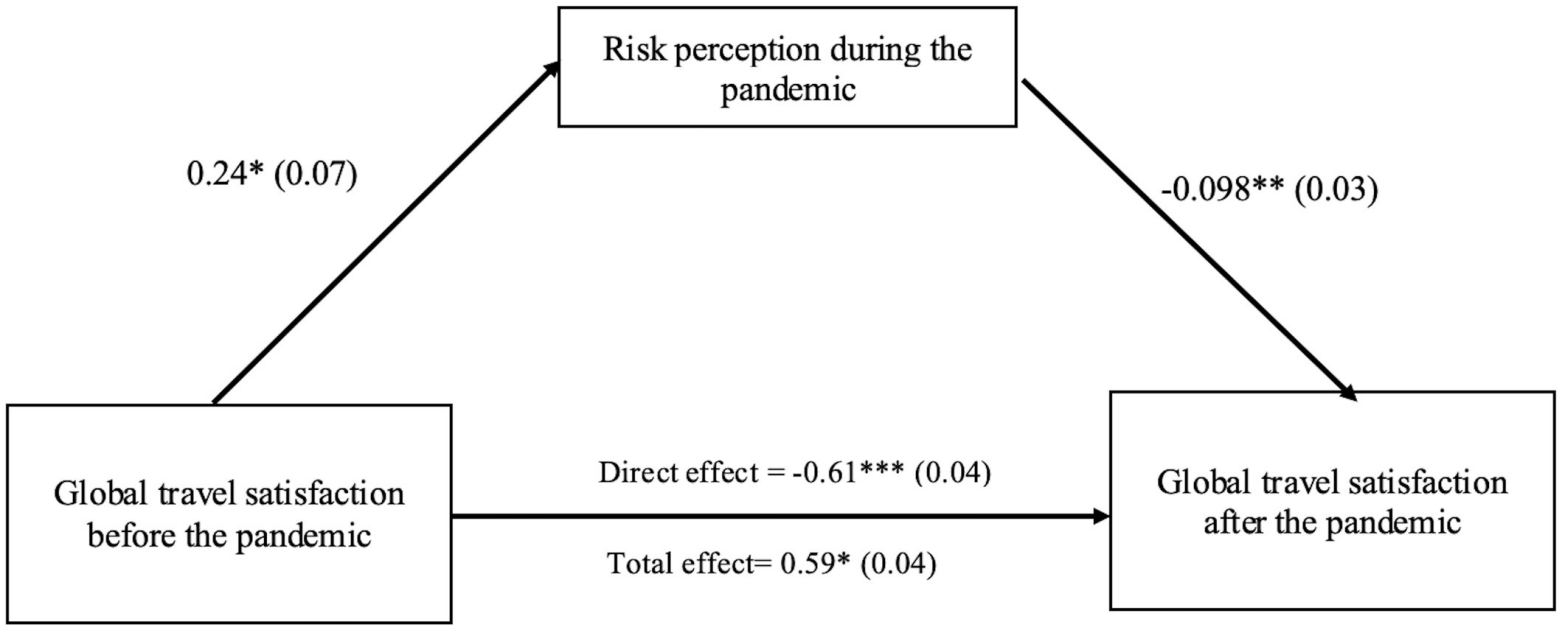
The conceptual model proposed to account for the relationship between global STS before the pandemic and global STS after the pandemic with the mediation effect of risk perception during the pandemic. Data values are unstandardized β regression coefficients, with standard errors shown in parentheses. p < .05*, p< .01* *, p< .001*** (two-tailed test).

A Sobel test was also used to verify the mediating effect of the risk perception during the pandemic between the global STS global score before the pandemic and the global STS global score after the pandemic. First, results of simple linear regression showed that the global STS before the pandemic was a statistically significant predictor of the global STS after the pandemic (b = 0.61, β = 60, t = 13.02, p < .001). Next, when the mediator, risk perception during the pandemic was entered in the regression analysis, the STS global score before the pandemic proved smaller (b = 0.59, β = 0.58, t = 12.44, p < .05). On the other hand, the mediator emerged as a significant predictor of the dependent variable (b = -.098, β = -0.12, t = -2.66, p < .01; 95% CI1 = -1.17 to -0.2). To further investigate the mediator, the Sobel test was utilized to examine if risk perception during the pandemic significantly mediated the relationship between the two variables. The results confirmed that the mediator significantly mediates the relationship between the STS global score before the pandemic and the STS global score after the pandemic (Z = 2.21, p = .02; see Fig 3).

## 4. Discussion

The present study examined the association between risk perception and travel satisfaction at COVID-19 pandemic outbreak related to the use of PT in Turin, Italy. Three different temporal scenarios (before the pandemic, during the pandemic and after the pandemic) were compared and the effect of gender and age was considered. Satisfaction with travel and risk perception in relation to the potential for the spread of COVID-19 infection, were considered two of the most important variables to understand potential impacts on mobility choices currently and into the future.

Regarding risk perception over time, the results of this study suggested that the COVID-19 pandemic emergency significantly changed the perception of possibly contracting infectious diseases when traveling on PT, both now and for the future. As such, risk perception related to COVID-19 contagion should be considered as an important predictor of travel choices in the future [62]. For this reason, the investigation of PT users’ expectations about their future perceptions may provide useful data for PT planning. In this study, the risk perception scale measured perceived risk in relation to the use of PT at the present moment. For the first subscale (the experience of traveling in a PT vehicle), about 30% of the participants expressed that they perceived high levels of risk at the present moment. For the second subscale (the experience of standing or moving in the adjacent/waiting spaces), the answers highlighted lower levels of perceived risk. These results suggest that the experience of traveling in a closed space, where proximity with other people may occur and over-crowding may arise, is subjectively perceived as riskier in comparison to staying or moving within the waiting spaces.

The results here presented highlighted a gender effect in risk perception, with women perceiving higher levels of risk, independent from the current pandemic emergency. Such results are consistent with literature on risk perception which shows gender-related effects [52,63], for example in the context of driving behaviour [22,25,26,64]. Women seem to perceive higher levels of risk than men regarding when they expect the virus will be clinically extinct, as highlighted by Lanciato et al. [53] who investigated risk perception in relation to COVID-19 although not in the PT context. Broadly speaking, women perhaps feel more consciously worried about the future as they are more able to anticipate their emotional responses for future adverse events [65,66]. This study highlighted a gender effect also regarding satisfaction with travel when imagining the future scenario, where women were found to perceive the travel experience as more negative than men. The authors postulate that these gender differences regarding risk perception and satisfaction with travel for the future are connected and explainable in terms of differences in personality traits (e.g. women are more conscientious) and in affective attitude of pandemic (e.g. women may engage in less positive future-oriented thinking) [54,67]. Furthermore, in line with Savadori et al. [54], it is possible that women are more likely to hold a communitarian view which may foster a negative appraisal of the pandemic, and a subsequent increase in risk perception.

Regarding travel satisfaction, the STS global scores collected in this study indicated that the subjective experience of wellbeing during PT travel has changed across the three temporal scenarios (before, during and after pandemic).

As far as age is concerned, the PT user group aged 36 to 50 years were identified as being the most satisfied with travel experiences across all three scenarios. This was followed by the group of older users aged 51 to 70 years and the younger group aged 18 to 35 years. This result is consistent with the literature that has found that users aged between 40 and 49 years are more likely to be satisfied with their journey [68] despite St-Louis et al. [69] and Ettema et al. [36] reported that older users experience higher satisfaction with travel.

When considering the scenario ‘during the pandemic’, no significant difference in terms of age or gender emerged in this study. This suggests that the disruptive experience of the COVID-19 pandemic is far more impactful than other socio-demographic variables.

Distinguishing the three dimensions measured by the STS, activation was the most negatively affected by the pandemic. The pandemic has increased respondents’ feelings of being worried or stressed about the prospect of using PT, and has increased a feeling of being hurried, especially for women.

The youngest age group in the study experienced the highest level of negative activation in the use of PT in the period before the pandemic. This result is consistent with other studies that have identified that traveling by PT is stressful for this age group [70]. In the case of Italy, this may be explained by the fact that the driver for using PT for many young people is a lack of alternatives. PT users in this younger age group experienced the most negative levels of satisfaction before and after the pandemic, aligning with the trends previously identified.

While activation remained high in users’ projections of future PT experience, satisfaction and pleasure levels were currently impacted by the pandemic but should return to previous levels in the future. Feelings of worry and stress related to the risk of contagion remain high in PT users’ future projections, perhaps influenced by the subjective well-being of urban PT users and their choice to use PT.

Interestingly, the data about travel satisfaction appears consistent with data about risk perception. In general, worry (measured by the STS in the activation dimension) can be understood as an emotional response to stress, potential risks or actual risks.

Indeed, the correlation between risk perception related to the COVID-19 pandemic and the activation dimension of the STS strengthens over time, highlighting that this pandemic has changed the parameters by which PT users evaluate the travel experience.

This result suggests that users’ perception of safety in relation to health is a critical factor influencing their overall satisfaction with PT travel. Such interpretation is strengthened by the results of the proposed mediation model: the global satisfaction with travel before the pandemic affects the global satisfaction with travel in the future through the partial mediating effects of risk perception during the pandemic, confirming that COVID-19 risk perception is nowadays a central issue when traveling by PT. Based on these findings, it can be concluded that risk perception plays an important role in the process of satisfaction evaluation that in turn may influence intentions and decisions about transport mode use.

In sum, the understanding of the travel satisfaction will increasingly pass through the perception of risk. In this sense, risk perception has become one of the determinants of the overall level of the travelers’ satisfaction. It also means that travelers are aware and take risks (and emotional feelings related to the risks) into account when evaluating the satisfaction with PT. These findings provide stakeholders with evidence about the importance to act for making PT safe and communicating it as such.

Despite this investigation regarded a relatively limited urban area in north Italy (Turin), the present study may be mostly representative of many urban/metropolitan areas of regions and countries featured by similar socioeconomic conditions and public transport density, that were affected by the pandemic contagion and the related measures to contain the infection during the first period of the COVID-19 outbreak. However, caution should be given when generalizing the results of this study worldwide. In fact, for example, the sample is not representative of all regions in Italy but only of those areas where the experience of the COVID-19 pandemic has been dramatic due to a far high number of infections. However, we may assume that risk perception and concern for contagion are similar throughout Italy, in part because when the Italian government extended the lockdown millions of citizens living in the Northern regions (Lombardy and Veneto above all) fled South on the last departing trains and buses. Two more limits need to be noted: first, this study did not adopt standardised measures to assess psychological distress or attitudes to risk-taking, instead we developed a questionnaire ad hoc that aimed to assess the constructs of interest. Second, some retrospective questions were used in order to collect travel satisfaction data. As such, the responses to these questions could be affected by psychological factors (e.g.: emotions and prejudices) involved in the recall of past experiences. The limitations of retrospective measurement of travel satisfaction and risk perception requires comparative data to be collected in the near future to enable more accurate longitudinal analyses.

However, despite these limitations, this study offers insights to better understand the complexity of psychological determinants in the use of PT in Italy, one of the countries hardest hit by the COVID-19 pandemic. It will be vital that stakeholders and decision makers responsible for PT policies consider the psychological processes investigated and analysed in this study.

There are several possible future lines of research arising from this work. One key area is examining changes in mobility patterns in advance and after the COVID-19 pandemic. Focussing on other socio-demographic variables (e.g.: education, socioeconomic status or employment status) and comparing different parts of Italy, would also provide further insights into the factors influencing risk perception and travel satisfaction during this pandemic. It would also be interesting to include other psychological variables (e.g.: attitude towards risk, social norms or personality traits) that may account for individual differences and/or cross-cultural differences in risk perception and travel satisfaction.

## 5. Conclusions

The present study results highlighted that COVID-19 influenced PT users’ risk perception and their travel satisfaction as measured by the STS, in particular the dimension of activation, both during the pandemic and in their projections about the future. Interestingly, especially women’s perceptions proved to be influenced by the pandemic outbreak. Moreover, according to our findings, it can be concluded that risk perception plays an important role in travel satisfaction evaluation that in turn may influence PT users’ decisions about travel choices.

Our results provide a possible interpretation of some of the determinants underlying the mobility shift recorded since the outbreak of the COVID-19 pandemic, highlighting the role of health-safety concerns and travel satisfaction over time. The increase in levels of activation and risk perception during and after the pandemic may indicate a permanent change in how people think about and the views they hold in relation to PT use.

Within this framework, this study explained that the COVID-19 outbreak—a period of uncertainty and risk—makes it relevant to assess factors- as risk perception- that influence travel satisfaction.

Notwithstanding its limitations, this research provides PT stakeholders with insights into certain psychological reactions to PT use in the pandemic (particularly in relation to age and gender variables) for consideration in the development of risk communication campaigns now and in the future.

These results highlight that the future of PT in cities, the satisfaction with travel by PT and the willingness of passengers to use it once the COVID-19 pandemic is over, are deeply influenced by the perception of risk of being infected by this contagious disease (especially for women). This needs to be a key consideration in the management of PT services and for communication with PT users in relation to safety measures adopted by the PT organisations and the behaviour required of users. The open challenge for the urban PT system in response to the public health security crisis is to meet the needs that have changed during these emergency situations. Communication will have a central role for such purpose, by enhancing passengers’ risk awareness and limiting unnecessary or disruptive beliefs and incorrect risk perception. Namely, effective and targeted communication, designed on the basis of scientific knowledge about specific risk perception and subjective levels of travel satisfaction, may contribute in empowering passengers’ knowledge about safety measures adopted by PT companies, and in increasing their vigilance and correct attitudes regarding healthy and safe behaviours.

On the other hand, PT stakeholders are requested to re-designing various aspects of PT services in order to avoid the spread of COVID-19 virus and other prospective diseases, so as to maintain a satisfactory level of safety thanks to preventive scientific-based measures (i.e.: providing adequate and regular disinfection of vehicles, increasing the frequency of service to limit overcrowding). Furthermore, strategic and operational re-planning regarding travel patterns is needed in a medium-long term perspective to provide robust responses in case of future outbreaks.

In conclusion, the impact of COVID-19 pandemic has represented an enormous challenge for the PT system and as a consequence for the issue of sustainable transportation. Nevertheless, it also presents an opportunity to deploy new safety measures and travel conditions that may in addition improve PT users’ safety perception and travel satisfaction more comprehensively.
